# Intelligence

**Published:** 1809-02

**Authors:** 


### INTELLIGENCE.

Pursuant to a Vote of the House of Commons, passed in the last Session, a NATIONAL VACCINE ESTABLISHMENT is now formed, by direction of HIS MAJESTY, for the purpose of promoting Vaccination throughout the United Kingdom, and is under the management of a board, consisting of the following Members:

Sir LUCAS PEPYS, Bart. President of the Royal College of Physicians in London; Dr. MAYO, Dr. HEBERDEN, Dr. SATTERLEY,

LEY, and Dr. BANCROFT, Censors of the College; GEORGE CHANDLER, Esq. Master, and ROBERT KEATE, Esq. and Sir CHARLES BLICKE, Governors of the Royal College of Surgeons in London.

*Director*, EDWARD JENNER, M. D. F. R. S.
*Assistant Director*, JAMES MOORE, Esq.
*Register*, Dr. HARVEY.
*Principal Vaccinator*, J. C. CARPUE, Esq.
*Vaccinators at the Stations*, CHARLES R. AIKIN, Esq. THOMAS HALLS, Esq. RICHARD LANE, Esq. EDWARD LEESE, Esq. S. SAWREY, Esq. J. P. VINCENT, Esq.
*Secretary*, CHARLES MURRAY, Esq.
House of the Establishment, No. 21, Leicester Square.

---

At the meeting of the Wernerian Natural History Society of Edinburgh, held on the 10th of December, the secretary read a communication from the Rev. JOHN FLEMING of Bressay, describing a narwhal or sea unicorn, of the species denominated *Le Narwhal Microcephule*, by La Cèpede, which had been lately cast on shore alive, at Weisdale Sound, in Mainland, the largest of the Shetland Islands. The description was accompanied by a correct drawing of the animal, which is to be engraved. At the same meeting Dr. OGILVY read a paper on the transition green stone of Fassnet, in East Lothian, which besides much valuable mineralogical information, contained a satisfactory answer to the query proposed some time ago by Professor Jameson, in regard to the geognostic relations of the rocks of this tract of country. The descriptions of the individual rocks, and their general and peculiar geognostic relations were detailed with ability; and the interest of the whole was increased by acute observations on the mode of examining and discriminating rocks—a subject of great importance, particularly to those who may be employed in examining the mineralogy of a country.

On the 12th and 19th of December, Mr. SOWERBY, author of British Mineralogy, delivered his long promised lecture on Chromatometry, at his house in Mead Place, near the Asylum. This lecture, the object of which is, to point out a new and ingenious mode of ascertaining the arrangement, mixture, and measure of prismatic tints, and to shew their correspondence with material colours, was accompanied by an exhibition, in which the prismatic tints were produced, as from the sun, moon, and stars; the sun as seen from the different planets, and a productor, sixty feet long, measuring an infinite series: also the material and prismatic tints, forming mixtures in union, with the effect as from candles and flambeaus, and a sort of prismatic illumination, with different lustres from metals, &c. The whole was elucidated by apparatus of a new and original kind, which promises to assist the philosopher in greatly extending our knowledge on this subject. Mr. Sowerby continues to repeat the lecture every Monday, and has announced a work, illustrative of his discoveries.

MEDICAL

### Small-Pox and Inoculation Hospitals, St. Pancras.

*Medical Supplement to the Report of the Committee.*

Your Physician has, in compliance with the order of the Special General Court in April last, refused inoculation of small-pox to all out-patients; but, notwithstanding the notice given of the above order in the public prints, and by a painted board at the entrance of the hospital avenue, the number of applications has been very considerable. It has been the business of your physician and apothecary to persuade the mothers, who have brought their children for this purpose, to suffer them to be vaccinated, and they have the satisfaction of reporting, that in fifty instances they have been successful. But more than two hundred have refused to attend to any argument, declaring, that if their children were not inoculated with small-pox, they shall take their chance.

Though the bills of mortality, especially for so short a period, cannot be considered sufficient evidence, yet as they have often been referred to on the subject of inoculation, your physician has thought it right to show, that hitherto there is at least no reason to suppose the casual disease was increased by the inoculation of out-patients.

The order for discontinuing that practice was made towards the end of April, and enforced in the beginning of May. From that time the number of deaths by small-pox., according to the bills of mortality, has gradually increased each succeeding month, so that the average for April and May was 42 ½ each, for October and November 141 ½ each

| | Inn Patients. | | | Out Patients. | | |
|---|---|---|---|---|---|---|
| | Small-Pox | Inoculated | Vaccinated | Vaccinated | Inoculated | Death by the Small-pox as reported by the Bills of Mortality. |
| January. | 7 | 21 | | 32 | 67 | 174 |
| February. | 9 | 19 | | 40 | 96 | 102 |
| March. | 9 | 29 | | 101 | 190 | 89 |
| April. | 4 | 36 | | 113 | 484 | 46 |
| May. | 4 | 25 | | 214 | 196* | 39 |
| June. | 15 | 16 | 1 | 117 | | 51 |
| July. | 14 | 10 | 2 | 53 | | 62 |
| August. | 3 | 10 | | 105 | | 78 |
| September. | 6 | 20 | | 156 | | 103 |
| October. | 10 | 23 | 3 | 159 | | 96 |
| November | 24 | 29 | | 114 | | 167 |

* To the 5th day of this month.

In the house, the number of patients received for the casual disease, has been stationary till the last two months, since which it has progressively increased. The number of fatal cases however is small, compared with the severity of some of them.

A patient

A patient under casual small-pox, has been received from a work-house at Bow. On inquiry, it appears that there are no less than thirty in that house, who have not gone through the disease. Your physician has thought it his duty to propose to the managers, the vaccination of the whole number, or the admission for inoculation of such as object to the former.

JOSEPH ADAMS, Physician.

H. C. WACHSELL, Apothecary.

---

Accounts from Brazil state, that the vaccine inoculation, first practised in St. Salvador, towards the close of 1804, has since been spread through all the provinces, by the orders of the prince regent. His royal highness appointed Dr. J. A. Barbosa to superintend and promote the new practice; and so beneficial have been its effects, that the small-pox, formerly very destructive there, has totally disappeared.

A species of wasp which builds its nests in trees has lately been observed in various parts of this country, and was frequently met with during the last summer in different parts of the West Riding of Yorkshire. It appears to be a new introduction, and is supposed to have been brought across the Atlantic into some of the ports on the western shore of the island, and is gradually spreading itself through the country. The trees on which the nests have been most frequently observed, are the gooseberry and currant, and an instance of it has been met with on the common elder, to which insects in general are averse. This species is smaller than the common wasp, but it is much less voracious, and less easily irritated.

Sir W. CLARGES, Bart. has constructed a life boat on an improved principle, the leading features of which, are, that she will not upset, sink, or be water logged; that she affords cabin room, and is like a man of war's launch, well built for rowing, the oars not on a curve, but nearly in a right line and low to the water, of which she draws little. The description of this boat is as follows:—Her length is thirty feet, her breadth ten, her depth three feet six inches. The space between her timbers is fitted up with pine wood; this is done with a view to prevent the water lodging there: the pine wood is well caulked and paid; she is buoyed up by eight metal cases, four on each side; these are water tight, and independent of each other. They will serve to buoy up six tons, but all the buoyant parts of the boat, taken collectively, will buoy up ten tons. The cases are securely decked over, and boarded at the sides with pine; there is a scuttle to each case, to put goods in; the edges are lined with baize; and over each scuttle, in the case, is one of wood of a larger size, the margin of which is lined in the same manner to exclude the water; between the cases are Norwegian balks, bolted to the bottom, fastened to each other by iron clamps, clamps, and decked over. The depth of her keel is nine inches below the starboard streak, the dead rising is four inches; her keel is narrow at the under part, and wide above, for the purpose of giving the timber a good bed, which will support the bolts, in case any necessity should arise, to encounter sand banks.—In sailing over a bar, or in places where the water is shallow, the rudder will, with ease, draw up even with the keel, and when in deep water, it will let down easily, and with equal facility. The forecastle of the boat forms a cabin ten feet wide, six feet long, and four feet deep, into which women, children, and disabled persons may be put; it is amply supplied with air by means of two copper ventilators; it is furnished besides with two grapnels, very proper to be thrown out on board a wreck, to ride by; the grapnel ropes will assist the sufferers to remove and escape from the wreck to the boat. She is likewise equipped with masts and sails, and is as manageable with them as any boat of her dimensions can possibly be; in a tempest, however, she must be dismasted, and rowed by fourteen men with oars sixteen feet long, double banked; the men are all fastened to the thwarts by ropes, and cannot be washed from their seats. In his observations on this boat, Sir William says, "Having stated the leading features of my boat, I need not dwell on a few secondary points, which, however, it would be improper not to mention: these are, her being provided with small ropes or lines fastened to hooks on the gun-wale, each having a piece of painted cork at the extremity, intended not only for persons who fall overboard or swim from a wreck, to see and catch hold of, but to tow any one for whom there may not be room in the boat; and her having a very powerful rudder. The copper cases, though affording additional security to those who choose to go to the expence, are no more a necessary point of my plan, than coppering her bottom. The wood work, if well executed and properly attended to, may be kept quite air tight.—If the assistance of cork were to be called in, it appears to me that it might be better applied than in the other boats, by filling the cases with cork jackets, to take to a crowded wreck; in going off to which the cases would not be wanted for any other purpose; the jackets would not be an incumbrance. Every one must be aware of the use of the side cabins or cases, for stowing valuable goods from a richly laden vessel. A boat of this kind, but somewhat smaller dimensions, would be exceedingly useful to ships on discovery, and indeed to any large vessels; as it would not only answer for wooding and watering, but is peculialy adapted for excursions up rivers or small inlets of the sea, or exploring clusters of islands. As a pleasure boat she answers extremely well; and with respect to her safety, I can say that I have sailed in her from Brighton, round the Cornish coast to Conway, in North Wales, without any accident, though we experienced some very dreadful weather on the voyage."

St. Tho-

St. Thomas's and Guy's Hospitals.

The Spring Course of Lectures at these contiguous Hospitals, will commence as usual the 1st of February, viz.

*At St. Thomas's*, Anatomy and Operations of Surgery, by Mr. Cline and Mr. Cooper.—Principles and Practice of Surgery, by Mr. Cooper.

*At Guy's*, Practice of Medicine, by Dr. Babington and Dr. Curry.—Chemistry, by Dr. Babington, Dr. Marcet, and Mr. Allen.—Experimental Philosophy, by Mr. Allen.—Theory of Medicine, and Materia Medica, by Dr. Curry and Dr. Cholmeley.—Midwifery, and Diseases of Women and Children, by Dr. Haighton.—Physiology, or Laws of the Animal Oeconomy, by Dr. Haighton.—Occasional Clinical Lectures on Select Medical Cases, by Dr. Babington, Dr. Curry, and Dr. Marcet.—Structure and Diseases of the Teeth, by Mr. Fox.

N. B. These several Lectures are so arranged that no two of them interfere in the hours of attendance; and the whole is calculated to form a complete Course of Medical and Chirurgical Instructions. Terms and other particulars may be learnt at the respective Hospitals.

Mr. Carpue will commence his Spring Course of Lectures on Anatomy, Surgery, &c. on Monday the 9th of January.—Particulars may be known by applying to Mr. Carpuè, No. 50, Dean Street, Soho.

Mr. Chevalier will commence his next Course of Lectures on the Principles and Operations of Surgery, on Monday, February 13, at eight o'clock in the evening, at his house in South Audley Street, Grosvenor Square.

Dr. Clutterbuck will begin his Spring Course of Lectures, on the Theory and Practice of Physic, Materia Medica, and Pharmaceutic Chemistry, early in January, at nine o'clock in the morning.—Particulars may be had on application, at No. 1, Crescent, New Bridge Street, Blackfriars.

Dr. Dennison and Dr. Byam Dennison will commence their second Course of Lectures, on the Theory and Practice of Midwifery, and the Diseases of Women and Children, on Monday, February 6, at eleven o'clock, at the London Hospital.—For particulars, apply to Mr. Price, apothecary to the Hospital, or at the Droctor's house, No. 37, Broad Street Buildings.

Dr. Hooper will commence a Course of Lectures, on the Theory and Practice of Physic, the Materia Medica, and Chemistry, on Wednesday, February 1, at his lecture room, in Cork Street, Burlington Gardens, at eight o'clock in the morning.—A prospectus with particulars may be had by applying to Dr. Hooper, at his house, No. 21, Saville Row.

---

Dr. Adams is preparing a new edition of Mr. Hunter's Treatise on the Venereal Disease. The object of the work is as follows: The text will be preserved without the smallest alteration. A long commentary will be added to the introductory chapter. Prefaces will precede, or commentaries follow, those chapters which have been thought obscure, or in which the doctrines have been disputed. The few points in which the commentator has differed from Mr. Hunter, (none of which materially affect the doctrine) will be stated, with the reasons for such difference.

That a work so necessary for every practitioner may be as extensively circulated as possible, the publishers have agreed to comprise the whole in an octavo volume, on as easy terms as the bulk of the materials will admit.

Dr. Kentish, of Bristol, has formed an establishment where the Faculty may order heat or cold, in any proportion, to be applied to a patient, either locally or generally; he has also published an Essay on Warm and Vapour Baths, with hints for a new mode of applying heat and cold for the cure of Diseases, and the preservation of health, illustrated by Cases.

Dr. Stock, of Bristol, has undertaken a life of the late Dr. Beddoes, with the approbation of his family and friends.

The long expected Reports of the Preventive Medical Institution at Bristol, have been left, by the late Dr. Beddoes, in some degree of forwardness. They will be completed and published, as soon as possible, by Mr. King and Dr. Stock. The former of these gentlemen has been Surgeon to the Institution from its first commencement; and the latter has been connected with it since the month of March, 1804.

Mr. Macartney is about to publish a small work, on the relations between external and internal parts, by which the situation of any important blood-vessel, nerve, &c. may be precisely ascertained in the living body. The subject will be illustrated by plates, and accompanied with practical and surgical observations.

