# Errata

**Published:** 1809-03

**Authors:** 


### ERRATA.

No. 120, p. 171, l. 2, *for* Robert Keate, *read* Thomas Keate, Esq.

